# Plantar pressure distribution and altered postural control in multibacillary leprosy patients

**DOI:** 10.1186/s12879-023-08749-0

**Published:** 2024-01-24

**Authors:** Alex Tadeu Viana Da Cruz Junior, Beatriz Helena Baldez Vasconcelos, Tatiana Generoso Campos Pinho Barroso, Givago Silva Souza, Luis Carlos Pereira Monteiro, Marília Brasil Xavier, Bianca Callegari

**Affiliations:** 1 Laboratory of Human Motricity Studies, Health Science Institute, Federal University of Para, Belém, Brazil; 2 Nucleous of Tropical Medicine, Federal University of Para, Belém, Brazil; 3 Federal University of Roraima, Boa Vista, Brazil; 4 Biological Science Institute, Federal University of Para, Belém, Brazil; 5 Biological and Health Sciences Center, State University of Pará (UEPA), Belém, Pará Brazil

**Keywords:** Plantar pressure, Postural control, Leprosy, Foot ulcer

## Abstract

**Background:**

Leprosy is a chronic infectious disease caused by *Mycobacterium leprae*, predominantly affecting the peripheral nerves, resulting in sensory and motor deficits in the feet. Foot ulcers and imbalances are frequent manifestations in leprosy, often correlating with diminished sensitivity. While clinical scales and monofilament esthesiometers are conventionally utilized to evaluate foot sensitivity and balance in these patients, their discriminatory power is limited and their effectiveness is greatly dependent on the examiner’s proficiency. In contrast, baropodometry and posturography offer a more comprehensive evaluation, aiming to preempt potential damage events. This study aimed was to assess the correlation between baropodometry and force plate measurements in leprosy patients and control participants, to improve the prevention and treatment of foot ulcers and complications associated with leprosy.

**Methodology:**

This cross-sectional study was conducted during 2022 and enrolled 39 participants (22 patients with multibacillary leprosy and 17 non-leprosy controls). Demographic data were collected, and a monofilament esthesiometer was used to assess sensory deficits. In addition, physical examinations and balance and plantar pressure tests were conducted.

The Student’s t-test was used to compare mean and maximum plantar pressures between groups. For most COP variables, a Mann-Whitney Wilcoxon test was used, except for AP amplitude which was analyzed with the Student’s t-test due to its normal distribution. The relationship between foot pressure and balance control was assessed using Spearman’s correlation, focusing on areas with significant pressure differences between groups.

**Principal findings:**

Leprosy patients showed increased pressure in forefoot areas (T1, M1, T2-T5, and M2) and decreased pressure in hindfoot regions (MH and LH) compared to controls. These patients also displayed higher AP and ML amplitudes, suggesting poorer COP control. Correlation analyses between the two groups revealed that foot plantar pressures significantly impact balance control. Specifically, increased T1 region pressures correlated with greater sway in balance tasks, while decreased MH region pressures were linked to reduced COP control.

**Conclusions/significance:**

The findings suggest a joint disturbance of plantar pressure distribution and static balance control in leprosy patients. These alterations may increase the risk of tissue injuries, including calluses and deformities, as well as falls.

**Supplementary Information:**

The online version contains supplementary material available at 10.1186/s12879-023-08749-0.

## Introduction

Leprosy is a chronic infectious disease caused by *Mycobacterium leprae*. It continues to pose a public health challenge in developing countries such as Brazil and India, with a prevalence rate of 1.05 per 10,000 inhabitants, in Brazil [1–3]. The disease has a specific tropism to peripheral nerves, with the ulnar and common peroneal nerves being commonly affected [4]. Sensory fiber lesions lead to loss of thermal and tactile sensitivity and muscle responses, resulting in foot deformities, paralysis, and muscle atrophy [5]. These factors increase susceptibility to changes in plantar pressure, leading to the development of plantar ulcers and affecting postural adjustments and control of static balance [6–8].

To assess foot sensitivity and balance, clinical scales and monofilaments esthesiometer are commonly used (i.e. Semmes-Weinstein monofilaments for peripheral neuropathy assessment) [9], but these tools have limited power of discrimination and are highly dependent on the skill of the examiner (Sensitivity ranged from 41 to 93% and specificity ranged from 68 to 100%) [6, 9–12].

Baropodometry and posturography are two technological tools that can complement foot sensitivity and balance assessments. Baropodometry uses a platform to provide information on the distribution of plantar pressure and can identify early changes in foot structure, which is crucial in preventing foot ulcers [13]. Posturography is performed on a force platform to measure center of pressure (COP) displacement, an indicator of balance control [14]. Baropodometers, designed for foot pressure assessment, are available in Static, for standing, and Dynamic types for walking, with costs ranging from a few thousand dollars to over $20,000 for advanced models. Conversely, Force Plates, used for biomechanics, gait, and balance, come in Uniaxial, Triaxial, and Instrumented Treadmill versions. Due to their intricate technology, these plates can cost anywhere from $5,000 to upwards of $50,000, especially when integrated with motion capture systems [15].

Integrating both tools can offer a more holistic evaluation and precise diagnosis, enhancing the prevention and management of foot ulcers and complications related to leprosy. Using adapted footwear is vital in preventing such ulcers [16]. Despite the importance of these technologies, research using baropodometry and posturography in leprosy patients is limited (Table 1).

**Table 1:** Research on the pathogenesis of plantar ulceration utilizing plantar pressure or COP measurement in Hansen’s disease

Reference, Year	Subject Number	Plantar Pressure measurements	COP measurements	Findings
Sabato et al., 1982 [5]	30 patients	Static balance pressure 6 areas	No	Association between presence of an ulcer and the foot ground pressure.
Greve et al., 1994 [17]	13 patients, 17 control	Static balance pressure 2 areas	No	Hemilateral asymmetry and increased pressure were associated with plantar ulcer
Bhatia & Patil, 1999 [18]	108 patients, 52 control	Dynamic peak pressure (walking) 10 areas	No	Hemilateral asymmetry and increased dynamic foot pressure were associated with plantar ulcer
Slim et al., 2012 [8]	39 patients	Dynamic peak pressure (walking) 4 areas	No	Highest pressure is associated with tactile sensitivity
van Schie et al., 2013 [19]	39 patients (with/ without ulceration)	Dynamic peak pressure (walking) Not divided in areas	No	Current and previous ulceration do not differ on barefoot pressure.
Condeiro et al., 2014 [20]	51 patients 20 controls	Static balance pressure 6 areas		Loss of protective sensitivity in multibacilar leprosy patients is predictive of plantar ulcers Plantar pressure peaks seem to be of greater importance in paucibacilar leprosy patients in ulcer prediction
Cordeiro et al., 2014 [16]	21 patients 11 controls	Static balance pressure 6 areas	ML and AP displacement	No differences in plantar pressure or COP measurements
Viveiro et al., 2017 [21]	34 patients 34 controls	No	Area, ML and AP velocity	Greater oscillation and velocity of COP in individuals with leprosy.
Tashiro et al., 2020 [22]	20 patients	Dynamic peak pressure (walking) 12 areas	No	Neuropathic foot avoids weight bearing in the foot area with sensory loss

Previous studies have mainly focused on plantar pressure measurements in leprosy patients for diagnostic purposes, such as comparing them to a control group [17, 18, 21, 23] or investigating the association between foot ulcers and plantar pressure [5, 17, 19]. Additionally, some studies have compared balance control between leprosy patients and controls [21, 23]. In comparing these two parameters within the same subjects, we hypothesized that an abnormal plantar pressure distribution as well as disturbances in the center of pressure (COP) would be observed in leprosy patients when compared to controls. We further hypothesized that these impairments would be correlated. Thus, in this study, our objective was to evaluate plantar pressure and COP control in leprosy patients and a control group and assess the correlation between baropodometry and force plate measurements. This approach is novel in leprosy research and aims to offer predictive insights to mitigate the risk of foot ulcers and falls in this population.

## Methods

### Subjects

This cross-sectional study was conducted, during 2022, at the Human Motricity Laboratory (LEMOH), located within the Institute of Health Sciences (ICS) of the Federal University of Pará, Brazil. All volunteers provided written informed consent to participate in the study, and all methods used were in accordance with the Declaration of Helsinki, as well as the Observational Studies in Epidemiology (STROBE) Statement.The Institute of Health Sciences Human Research Ethics Committee approved the current investigation (report #5468074, CAAE54882321.8.0000.5172).

The study utilized a non-probabilistic purposive sampling method and comprised 39 participants. These individuals were categorized into two groups: 22 multibacillary leprosy patients and 17 non-leprosy volunteers forming the control group. These participants were diagnosed and selected from the Tropical Diseases Center at the Federal University of Pará. The inclusion criteria involved diagnosing leprosy infection through a general clinical and dermato-neurological evaluation, adhering to the criteria set by the World Health Organization. To determine the inclusion criteria, tests were applied to detect changes in skin sensitivity or impairment in peripheral nerves, encompassing sensory, motor, and autonomic nerves. For diagnosing leprosy infection and assessing the inclusion criteria, tests targeting skin sensitivity changes and peripheral nerve impairment were employed. The specific tests were general clinical and dermato-neurological evaluations, as guided by the World Health Organization’s established criteria. These evaluations and tests were carried out by expert professionals from the Tropical Diseases Center at the Federal University of Pará. These professionals possess specialized expertise in leprosy and are adept in identifying sensory, motor, and autonomic nerve disturbances characteristic of the disease.

Conversely, the study’s exclusion criteria comprised patients incapable of standing unaided or those with conditions impacting plantar pressure, such as other neurological ailments, amputations, diabetes, rheumatic diseases, or a familial history suggesting hereditary neuropathy. Additionally, patients with conditions like hepatitis infections, Human T-Cell Lymphotropic Virus Type, HIV, peripheral vestibular syndrome, or physiological states like pregnancy, which might modify regular plantar pressure, were also ruled out.

### Procedures

#### Demographic and clinical measurements

All subjects underwent the same initial assessment protocol to ensure the similarity of the groups, except for the diagnosis, which included gathering demographic data such as age, height, weight, and sex, as well as an additional assessment using a monofilament esthesiometer to measure the presence of sensory déficits.

The tactile sensitivity of the foot plantar skin was investigated using Semmes Weinstein monofilaments. A set of 5 monofilaments with varying force levels of 0.2, 2, 4, 10, and 300 g was utilized. The participants were lightly touched with the monofilaments in 8 different areas of the foot, including 6 areas in the forefoot, 1 in the midfoot, and 1 in the hindfoot, and asked whether they felt the monofilament touching their skin. Each monofilament was applied three times, starting with the lightest one. The sensitivity threshold was determined as the lightest monofilament identified by the subject. If the threshold was higher than 0.2 g, the skin tactile sensitivity in that area was considered to be altered. Data was reported as the value of the monofilament that was first perceived in at least 50% of all areas evaluated. The researchers also conducted a physical examination to identify neural complications, such as nerve thickening, pain, shock, edema, or adherence. Following the initial assessments, both groups underwent balance and plantar pressure tests.

#### Plantar pressure measurements

The barefoot plantar pressure measurements were obtained using a Capacitive Platform EPS/R1: EPS (Electronic Pedo-Scanner), from Loran Engineering, Castel Maggiore Bologna, Italy. This capacitive platform is a pressure-sensitive plate that contains 2,224 sensors distributed over 48 cm², with a sampling frequency of 50 Hz, and connected to a computer with Biomech software. The technology aims to identify abnormal pressure patterns.

The evaluation of all subjects was carried out under constant environmental lighting and sound conditions. Static analyses were performed with bare feet in bipedal support, with feet positioned at a distance proportional to the width of the shoulders and with arms extended along the right and left body. The subjects directed their gaze to a white circular target painted on the wall located 1 m away. Plantar pressure was recorded in three sessions, each lasting 1 min, conducted with eyes open. There was a rest interval of 30 s between consecutive recording sessions. Mean values of the three sessions were used for further data analysis [24].

The variables obtained were the mean and maximum values of plantar pressure were assessed in ten distinct regions of each foot: T1 (hallux), T2, T3, T4, T5 (other toes), M1 (first metatarsal), M2 (second metatarsal), M3 (third metatarsal), M4 (fourth metatarsal), M5 (fifth metatarsal), MF (midfoot), MH (medial heel), and LH (lateral heel). For each trial, pressures were estimated as percentages of total foot pressure for each subject.

#### COP measurements

The posturographic signals were recorded by a force platform (Biomec 400, EMG System do Brasil, LTDA, SP), sampling rate of 100 Hz using a equipped with load sensors distributed over an area of 50 cm², and connected to a computer running Biomec software (EMG System do Brasil, Ltda., SP, Brazil). The sensitivity of each sensor was certified to be 0.0015% for a maximum load of 1000 N. The system employed a 16-bit analog-to-digital converter and a 0–50 Hz bandpass filter.

The volunteers were positioned in an orthostatic stance on the force platform and instructed to keep their arms relaxed at their sides. Environmental illumination and sound conditions were kept constant during the evaluation of all subjects. The test consisted of three trials, each lasting for 1 min, with the subjects instructed to keep their eyes open and gaze at a target with a black cross painted on the wall 1 m away. Two consecutive recording sessions were separated by a period of rest lasting between 30 and 60 s. The displacements of the center of pressure (COP) in the anterior-posterior (AP) and mediolateral (ML) planes as a function of time were recorded. The time series data were analyzed in the time domain [25], disregarding the first 5 s since this is considered the adaptation period [26], using the following parameters:

(i) RMS amplitude of the stabilograms in the AP and ML (AP and ML amplitudes), in centimeters (Eq. 1).

1 $${RMS}_{amplitude}=\sqrt{\sum\nolimits _{i=1}^{n}{\left({X}_{\text{i}}\right)}^{2}}$$

where ${\text{X}}_{i}$ is the reading of the device in the moment of the recording, $\text{n}$ is the total number of readings of the recording in the anteroposterior or mediolateral axes.

(ii) Total deviation of the statokinesiogram (DT) represents the length of the trajectory of the center of pressure over the base of support, in centimeters (Eq. 2).

2 $${RMS}_{amplitude}=\sum \sqrt{{\left({X}_{\text{A}\text{P}}\right)}^{2}+{\left({X}_{\text{M}\text{L}}\right)}^{2}}$$

where ${\text{X}}_{AP}$ is the vector of readings in the anteroposterior axes, ${\text{X}}_{ML}$ is is the vector of readings in the mediolateral axes

(iii) Statokinesiogram deviation area (AR), which is also an indicator of the two-dimensional deviation of the center of pressure over the base of support, in square centimeters (Eqs. 3 and 4).

3 $$\left[vec,val\right]=eig\left(cov\left({X}_{AP},{X}_{ML}\right)\right)$$

4 $$area=pi*prod(2.4478\times \sqrt{svd\left(val\right)})$$

Where $eig$ is MATLAB function to calculate the eigenvalues ($val$) and eigenvectors ($vec$) of the covariance of the vectors of readings in the anteroposterior and mediolateral axis calculated using the MATLAB function $cov$. $svd$ is a MATLAB function to proceed the single value decomposition of the $val$. $prod$is a MATLAB function to calculate the product of the array elements.

#### Statistics

The study estimated the total sample size in a pilot study with ten individuals using BioEstat (version 5.3, Amazonas, Brazil), with an alpha of 0.05 and a power of 0.90. Based on a for T1 mean pressure of 5.04 ± 1.33 (control group) and 6.90 ± 1.82 (leprosy group), it was determined that a minimum of 15 patients were needed in each group. The effect size was established using Cohen’s “d,” with a minimum effect size of 0.50 (average effect).The normality of the distribution for the variables was assessed using the D’Agostino test. To compare the mean and maximum plantar pressures between the groups, a Student’s t-test was applied. For comparing the COP variables, a Mann-Whitney Wilcoxon test (non-parametric) was performed, except for AP amplitude, which exhibited a normal distribution, and hence a Student’s t-test was employed for this comparison. The leprosy group was divided into two subgroups based on the extent of sensitivity loss: a low loss group with sensitivity to a 2 g monofilament and a high loss group with sensitivity to 4.0 and 10 g monofilaments. A Student’s t-test was applied to compare the differences between these subgroups. The association between foot pressure and balance control was assessed using linear mixed-effects models (LMMs) [27]. All LMMs incorporated the grouping factor (control and leprosy) as a random effect to account for the clustered structure in the observations. To verify model assumptions, we conduct visual inspection on models’ residuals followed by a Shapiro-Wilk normality test and a Bartlett test of homogeneity of variance [27]. All statistical analyses were conducted using GraphPad Prism (version 10.0.0, GraphPad Software, Boston, Massachusetts USA, www.graphpad.com), and the level of significance was set at *p* < 0.05.

## Results

### Demographic and clinical evaluation

The assessment of feet tactile sensitivity was diminished (sensitivity to the 2 g monofilament or above) in 20 of 22 patients. Demographic and Clinical data are available in Table 2.

**Table 2:** Demographic data of the subjects (Means ± standard deviations)

	Control	Leprosy	*P*-value
**Sex**	8 M, 9 F	8 M, 14 F	0.23
**Age (years)**	53.9 ± 2.2	51.2 ± 8.1	0.53
**Weight (Kg)**	66.23 ± 8.16	64.47 ± 8.84	0.24
**Height (m)**	1.61 ± 0.07	1.61 ± 0.06	0.91
**Sensitivity**			
** 0.02 g**	8 M, 9 F	1 M, 1 F	
** 2 g**	0 M, 0 F	5 M, 6 F	
** 4 g**	0 M, 0 F	0 M, 1 F	
** 10 g**	0 M, 0 F	4 M, 4 F	

*M* Male, *F* Female

During the physical examination, 22 patients displayed neural injuries. Fifteen had issues with the posterior tibial nerve, resulting in sensory and motor complications, and 10 with the common peroneal nerve. Specifically, 7 patients had changes in both nerves. Lower limb inspections found 2 with mobile clawed fingers (fourth and fifth toe), 9 with dry skin, 10 with hyperkeratosis, and 10 with hypotrophy (2 showed prominence of the head of the 1st metatarsal, and 10 presented decreases in muscle strength). Twelve patients reported pain, while five experienced paresthesia. None had recent ulceration or amputations.

### Plantar pressures

**Fig. 1:**
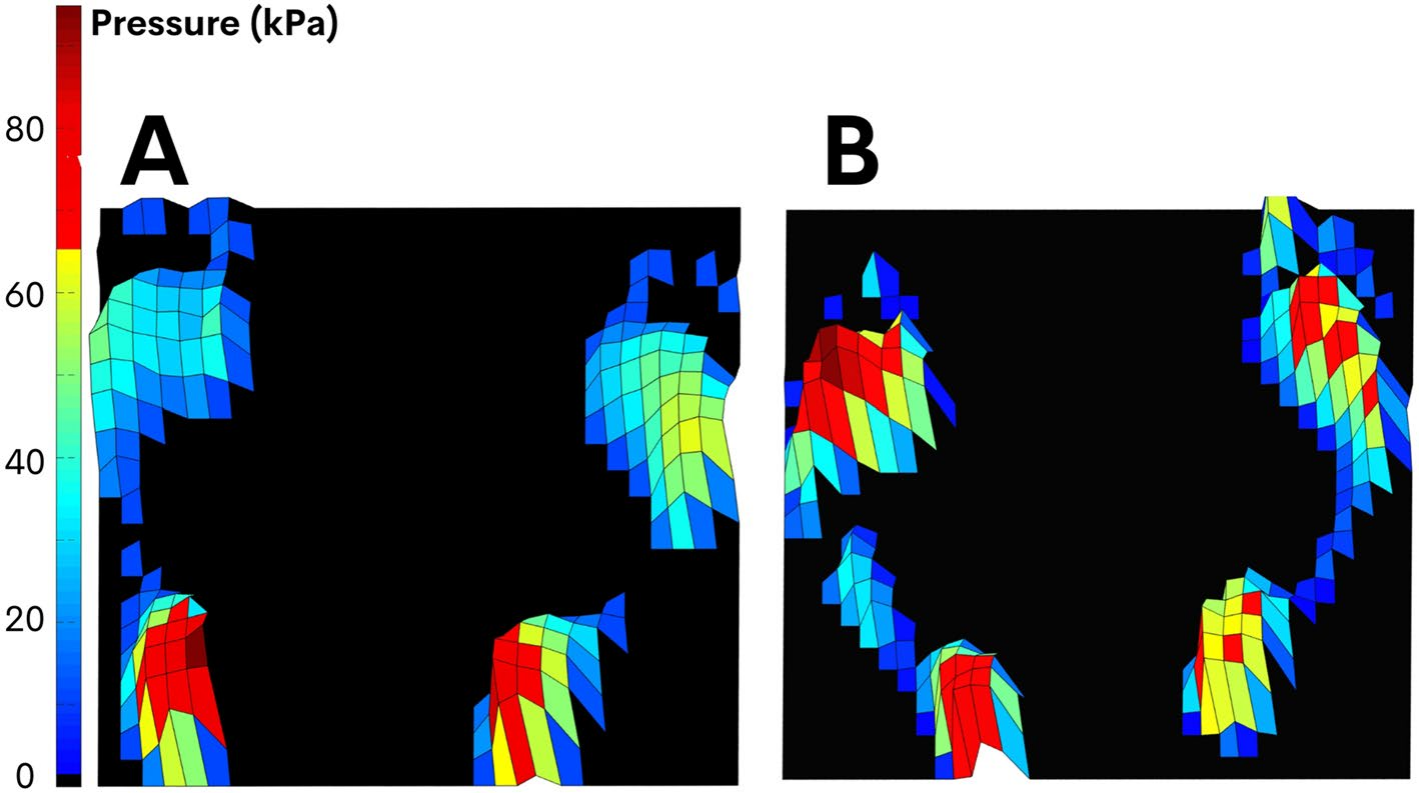
Mean peak pressure (kPa) of a typical subject from control (**A**) and leprosy group (**B**). Note high values of pressure in forefoot region in **B**

The mean and maximum foot pressures were measured and compared between the leprosy patient group and the control group. Figure 1 depict a typical subject from each group.

**Fig. 2:**
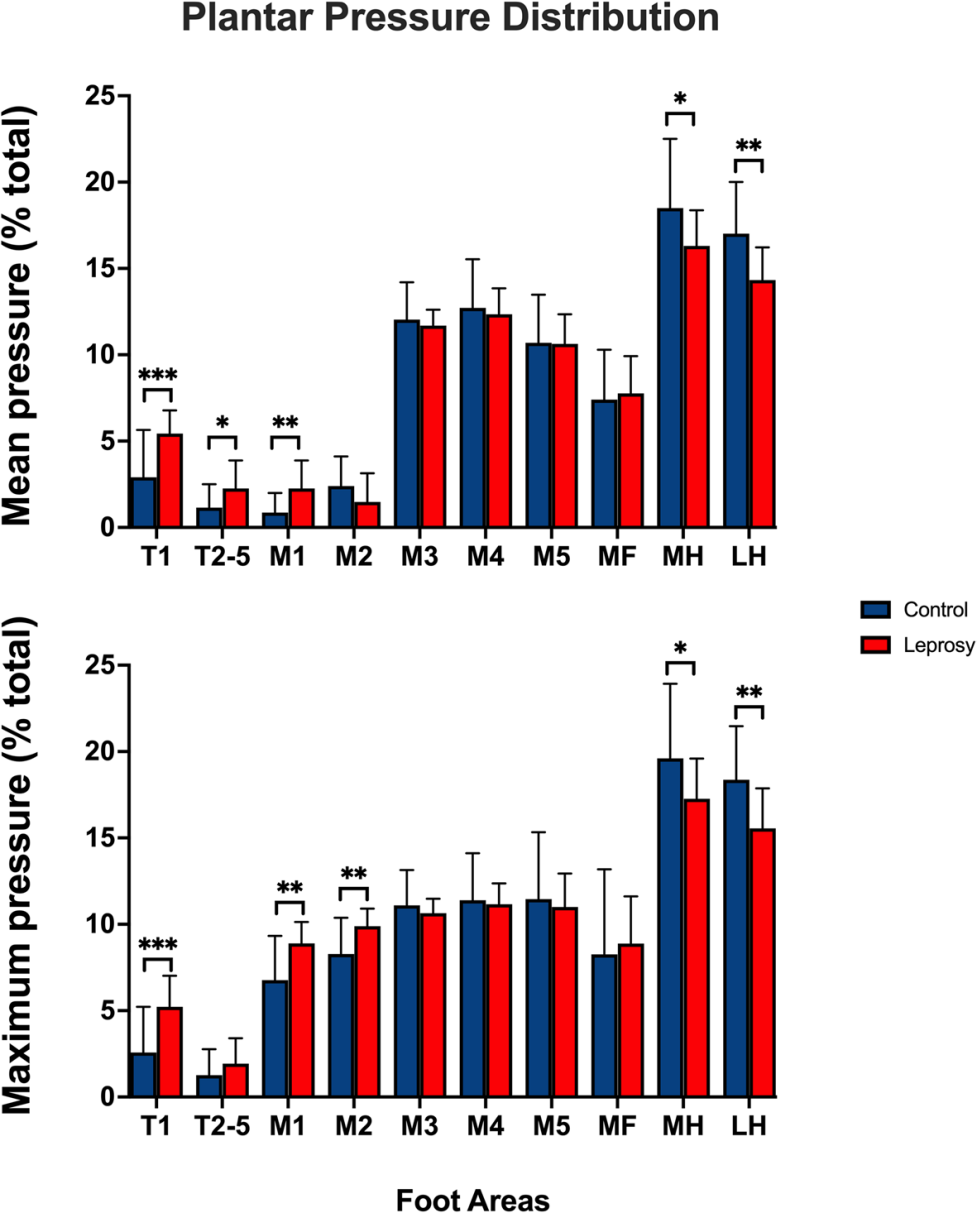
Comparison of plantar pressure distribution between control (blue bars) and leprosy patients (red bars). Means and standard deviations of the mean and maximum pressure values obtained in the open eye condition in both groups. *p < 0.05 **p < 0.001 and ***p < 0.0001

The results indicated that the leprosy patients discharged greater pressure in the forefoot regions and lower pressure in the hindfoot region, compared to the control group (Fig. 2). Specifically, in the forefoot region, leprosy patients exhibited higher mean and maximum foot pressures at T1 and M1 when compared to the control group (T1 mean: t[37] = 3.801, *p* = 0.0005; T1 maximum: t[37] = 3.719, *p* = 0.0007; M1 mean: t[37] = 3.023, *p* = 0.0045; M1 maximum: t[37] = 3.412, *p* = 0.0016). Moreover, the leprosy patients had higher mean and maximum pressure in T2-T5 and M2 regions (T2-T5 mean: t[37] = 2.275, *p* = 0.0288; M2 maximum: t[37] = 3.175, *p* = 0.0030). In contrast, both MH and LH areas in the hindfoot region presented lower mean and maximum foot pressures in leprosy patients (MH mean: t[37] = 2.213, *p* = 0.0331; MH maximum: t[37] = 2.164, *p* = 0.0370; LH mean: t[37] = 3.416, *p* = 0.0016; LH maximum: t[37] = 3.247, df *p* = 0.0025).

### COP results

**Fig. 3:**
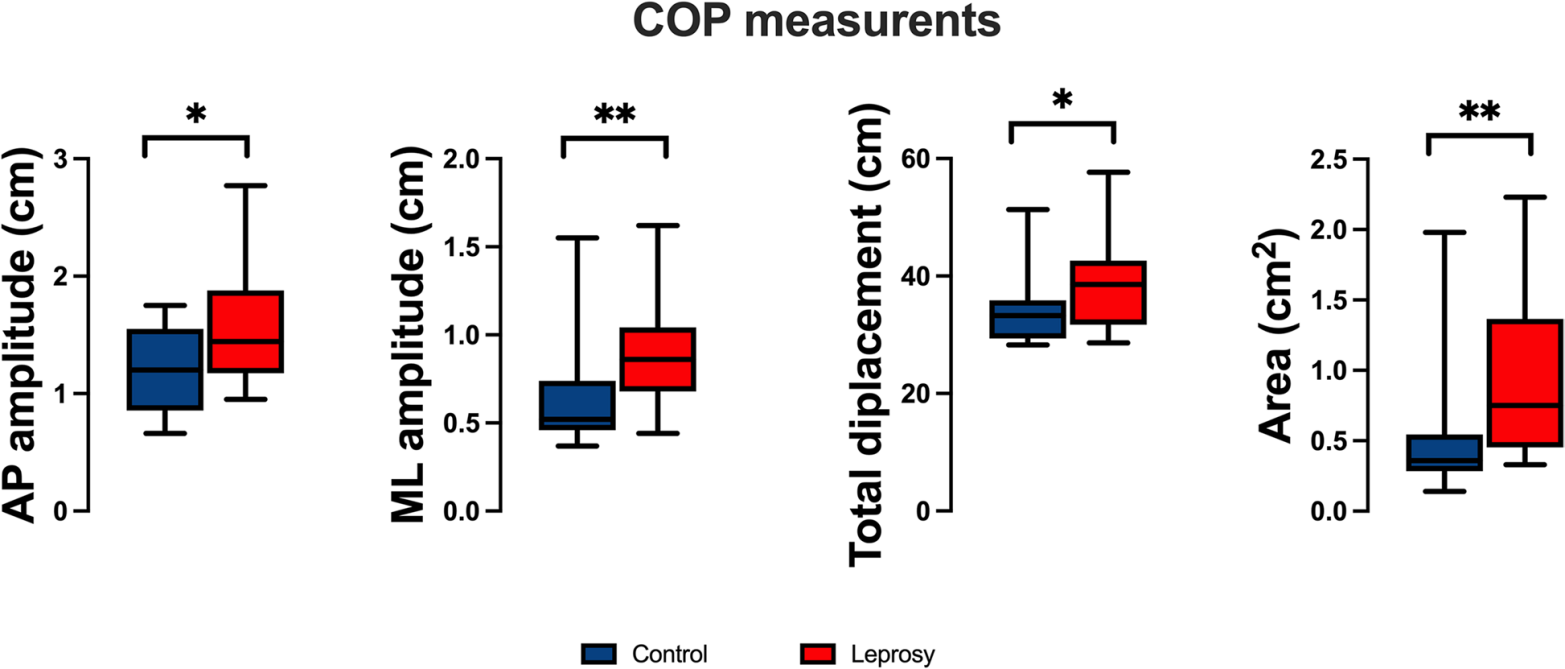
Comparison of the posturography estimated with open eye condition. ML: mediolateral; AP: anteroposterior; COP: center of pressure. Control group in blue and Leprosis in red). *p < 0.05 and **p < 0.001

Leprosy patients exhibited significantly higher AP amplitude (t[37] = 2.490, *p* = 0.0174), ML amplitude (U = 78, *p* = 0.0015), TD (U = 115, *p* = 0.0419), and AR (U = 78.5, *p* = 0.0016) when compared to the control group (see Fig. 3).

#### Comparison among leprosy patients with varying degrees of sensitivity loss

When comparing subgroups within the leprosy patient cohort, it was observed that those with greater sensitivity loss exerted higher pressure in the forefoot regions, specifically in T1 and T2-T5 areas (as shown in Table 3).

**Table 3:** Comparison between leprosy patients based on sensory impairment

Sensitivity Loss	T1 (%)	T2-5 (%)	M1 (%)	MH (%)	LH (%)	AP (cm)	ML (cm)	TD (cm)	AR (cm2)
**Low (2.0 g monofilament)**	4.62* (1.03)	1.77* (1.82)	1.76* (1.18)	16.80 (1.28)	14.92 (1.22)	1.51 (0.50)	0.90 (0.210)	38 (8.78)	0.89 (0.63)
**High (> 4.0 g monofilment)**	6.62 (0.66)	2.97 (1.97)	2.96 (1.97)	15.60 (2.78)	13.47 (2.41)	1.68 (0.58)	0.88 (0.21)	39.64 (6.14)	0.99 (0.57)

Mean and SD values for Leprosy sub groups

*p < 0.05

#### Correlation between plantar pressures and balance control parameters

In the correlation analysis that included both the control and leprosy groups, moderate and significant correlations were observed between the center of pressure (COP) parameters and foot plantar pressures (Table 4). Specifically, positive correlations were identified between the area pressures of the T1 region and the COP parameters, namely the anterior-posterior (AP) amplitude. Conversely, we found negative correlations between the area pressures of the MH region of the foot and the ML amplitude and area of the COP parameters and LH region and TD of the COP.

**Table 4:** Correlation between the mean plantar pressure and COP measurements

		T1	T2-T5	M1	MH	LH
**AP**	**general**	0.74 (0.02*)	0.04 (0.48)	-0.01 (0.86)	-0.01 (0.69)	-0.01 (0.65)
**ML**	**general**	0.30 (0.32	0.02 (0.57)	-0.02 (0.56)	-0.03 (0.04*)	-0.02 (0.25)
**TD**	**general**	0.39(0.46)	0.45 (0.55)	-0.14 (0.86)	-0.28 (0.045)	-0.98 (0.03*)
**AR**	**general**	0.04 (0.32)	0.03(0.55)	-0.03 (0.59)	-0.07 (0.01*)	-0.02 (0.54)

Linear mixed-effects model ß coefficient and *p*- values

*ML* Mediolateral, *AP* Anteroposterior, *TD* Total displacement

*p < 0.05

## Discussion

The present study aimed to investigate the relationship between plantar pressure distribution and static balance control in individuals affected by multibacillary leprosy without history of ulceration or neuropathy. Our results confirmed our hypothesis that leprosy patients would exhibit alterations in both COP and plantar pressure distribution. Specifically, we found that leprosy patients displayed increased pressures in the forefoot and reduced pressures in the hindfoot when compared to the control group. In addition, our posturography results revealed that leprosy patients showed higher body oscillations compared to the control group.

Our second hypothesis, which posited a correlation between plantar pressure distribution and static balance control, was also confirmed. We found that participants with higher pressures in the forefoot areas presented higher amplitudes in COP parameters, indicating a moderate correlation. Conversely, higher pressure in the hindfoot was correlated with lower amplitudes in COP parameters. Overall, these findings suggest a joint disturbance of plantar pressure distribution and static balance control in individuals affected by leprosy.

Our study confirmed previous findings that patients with leprosy shift their weight to the forefoot, leading to increased plantar pressures in this region and decreased pressures in the hindfoot area, compared to the control group [8, 17, 19]. This trend is consistent with previous research showing that patients with sensory loss tend to unload more weight from the affected areas, resulting in reduced load-bearing capacity In our study, 11 patients were identified as having impaired local sensitivity to pressure and/or loss of protective sensitivity, as indicated by their failure to respond to the 10 g monofilament test. Moreover, it was observed that these patientes with greater sensitivity loss exerted higher pressure in the forefoot regions compared to those preserved local sensitivity. This finding suggests that the impaired local sensitivity to pressure and/or loss of protective sensitivity may contribute to the higher plantar pressures observed in the group of patients with leprosy compared to the control group. In discussing the intricacies of plantar pressure distribution and control of the center of pressure (COP), it’s paramount to consider the degree of physical disability present in the individuals. Previous studies have highlighted that the severity and type of physical disability can significantly influence the dynamics of foot pressure and stability during standing or walking tasks. For individuals with pronounced physical disabilities, alterations in the plantar pressure distribution can lead to task imbalances, thereby affecting their overall postural control and stability [28]. Consequently, when assessing and interpreting plantar pressure and COP control in our study population, accounting for any underlying physical disabilities becomes essential to understand the broader context of how these factors interact and impact an individual’s gait and balance. The current study extends the existing literature by being the first to investigate both baropodometry and posturography in the same group of leprosy patients, thus providing a more comprehensive understanding of the relationship between plantar pressure distribution and postural control in this population.

In the present study, our findings suggest that leprosy patients exhibit significantly increased center of pressure (COP) variables. Specifically, we observed greater anterior-posterior (AP) and medial-lateral (ML) amplitudes, as well as increased area and total displacement of COP. These results suggest that leprosy patients experience more difficulty in maintaining their center of mass within the limits of stability, which may impact their overall balance control. To the best of our knowledge, few studies have investigated COP control in the leprosy population, and none have associated plantar pressure distribution with postural control. However, Viveiro et al. (2017) recruited both healthy and leprosy subjects and observed differences in COP behavior between the two groups during static evaluation on force platforms. Consistent with our results, the leprosy group showed greater COP oscillation in both AP and ML axes [21]. Cordeiro et al. [16] also compared sensory loss and balance deficits between healthy subjects and leprosy patients and reported a higher average of COP displacements in multibacillary leprosy patients when compared to controls. These findings collectively suggest that leprosy patients may be at risk of balance disturbances due to their altered plantar pressure distribution and increased COP variability.

Based on previous reports, it has been observed that individuals diagnosed with leprosy exhibit modified plantar pressures when compared to healthy individuals. This alteration in plantar pressures, combined with increased displacements of the center of pressure (COP), renders leprosy patients more susceptible to plantar injuries and falls. In this current investigation, we have demonstrated a clear correlation between anteriorization of weight bearing and a deficit in postural control. It stands to reason that subjects who display higher levels of pressure in the forefoot region would also exhibit greater COP oscillations. Additionally, the normal pattern of weight distribution involves greater weight bearing in the hindfoot region. Thus, we can deduce that leprosy disease, resulting from sensory loss, induces an imbalance in weight bearing which in turn alters patterns of plantar pressure and directly impairs control of the center of mass, as evidenced by COP oscillations.

The present study has several limitations. Firstly, detailing the location of the pain, its grading at the time of the test, and any associated pain relief medication usage would have enriched our findings. Thus, further studies are warranted to evaluate and intervene in the functional impairments experienced by this vulnerable population. The current results provide insight into the alterations experienced by this population and may help to inform behaviors that mitigate the risks to which they are particularly susceptible.

In summary, our findings demonstrate that leprosy patients without plantar ulcers display heightened plantar pressure in the forefoot region, which positively correlates with increased COP variables. As such, early assessment of plantar pressure distribution and static balance control may be an important screening tool for identifying postural disturbances that could increase the risk of tissue injuries, including calluses and deformities, as well as falls.

### Supplementary Information

**Additional file 1.** Datasheet.

## Data Availability

The datasets generated during and/or analyzed during the current study are available as supplementary files.
